# Cross-sectional study of Middle East respiratory syndrome coronavirus in humans and dromedary camels in Diyala, Iraq

**DOI:** 10.55730/1300-0144.5390

**Published:** 2022-01-04

**Authors:** Abdulrazak Shafiq HASAN, Kareem Saadoun ALI, Mohammad Kassem SALEH

**Affiliations:** 1 Department of Medical Microbiology, College of Medicine, University of Diyala, Diyala, Iraq; 2 Department of Veterinary Microbiology, College of Veterinary Medicine, University of Diyala, Diyala, Iraq; 3 Department of Family and Community Medicine, College of Medicine, University of Diyala, Diyala, Iraq

**Keywords:** MERS-CoV, anti-MERS-CoV Ab, Hajj-associated viral infection

## Abstract

**Background/aim:**

Middle East respiratory syndrome (MERS) is a zoonotic viral disease transmitted from dromedaries to humans. To date, more than 1500 cases of MERS have been reported and 80% of all cases have occurred in the Kingdom of Saudi Arabia (KSA).

This cross-sectional study was carried out to figure out the rate of infection among humans and dromedary camels and to explore the risk factors.

**Materials and methods:**

This study was conducted in Diyala Province, Iraq for the period from August 2017 to October 2018. Human subjects included 90 participants; 34 (37.8%) were females and 56 (62.2%) were males. Additionally, 90 dromedary camels were also included, 50 (55.6%) males and 40 (44.4%) females. Serum samples from subjects were collected and tested for the presence of anti-MERS-coronavirus (CoV) immunoglobulin g (IgG).

**Results:**

The results revealed that 46 (51.1%) of human subjects were positive for anti-MERS-CoV IgG, (95% confidence interval (CI) for the prevalence rate 40.9–61.3) with a mean titer of anti-MERS-CoV IgG antibodies (Ab) of 81.2 U/mL. The anti-MERS-CoV IgG positivity rate was insignificantly higher, but the mean of anti-MERS-CoV IgG titer was significantly higher among females (p = 0.12 and p < 0.004, respectively). Furthermore, the anti-MERS-CoV IgG positivity rate and Ab titer were significantly higher among those people who visited KSA for Hajj or Umrah (p < 0.001 and p < 0.001, respectively). In camels, 81 (90.0%) were positive for anti-MERS-CoV IgG, (95% CI for the prevalence rate 82.5–94.9), with a mean titer of 99.8 U/mL.

**Conclusion:**

The MERS-CoV infection rate was high among both Iraqi humans and dromedary camels. Further confirmatory studies are needed, and setting up of national precaution program is essential.

## 1. Introduction

The MERS-CoV infection first emerged in the KSA in 2012 and has spread to 27 countries. However, 80% of all cases have occurred in KSA and the largest outbreak outside KSA occurred in South Korea in 2015 [1,2]. Dromedary camels were the only animal species for which there was convincing evidence that it was a host species for MERS-CoV and hence a potential source of human infection [3,4]. Furthermore, epidemiologic investigations identified dromedary camels as the likely source of zoonotic transmission of MERS-CoV to humans [5,6]. Of note, it was well documented that the seroprevalence of MERS-CoV antibodies was very high reaching up to 100% in dromedary camels in the Arabian Peninsula as well as many other African and Asian countries [7–10].

The pattern of shedding and propensity for the upper respiratory tract infections (URTIs) in dromedary camels may help explain the lack of systemic illness among naturally infected camels and the means of efficient camel-to-camel and camel-to-human transmission [6,11]. MERS-CoV is mainly acquired in dromedaries when they are less than 1 year of age, and the proportion of seropositivity increases with age to a seroprevalence of 100% in adult dromedaries [12,13].

In humans, MERS is mostly known as a lower respiratory tract disease involving fever, cough, breathing difficulties and pneumonia that may progress to acute respiratory distress syndrome, multiorgan failure, and death in 20% to 40% of cases (the percentage increases with age); however, MERS-CoV has also been detected in mild and influenza-like illnesses and in those with no signs or symptoms [5,14]. The median incubation period was 5.2 days (1.9 to 14.7), and the serial interval was 7.6 days (2.5 to 23.1) [15]. Most confirmed cases so far were part of MERS-CoV clusters in hospital settings, affecting mainly middle-aged men and patients with a preexisting chronic morbidities or immunosuppressed status and the male to female ratio was 1.7:1 [16,17].

Clinically, it has been reported that the predominant comorbidities included hypertension, diabetes, respiratory, and renal disease, and the fever was the most common complaint [15]. Among other reported risk factors were healthcare workers and higher exposure occupations that bring people into close contact with camels which might be the source of infection for patients with confirmed MERS with no previous exposure to camels [18,19]. A systematic review and metaanalysis studies investigated people from different countries who visited the KSA for Hajj pilgrimage or Umrah had denied any infection by MERS-CoV in their citizens after their return form KSA [20–22]. There are ongoing efforts to develop MERS-CoV vaccines for humans and dromedary camels [23].

## 2. Materials and methods

This study was conducted in Diyala Province, Iraq for the period from mid-August 2017 to the end of October 2018. Human subjects included 90 participants who were subdivided into 3 groups; 40 individuals had close contact with camels, 20 were normal healthy individuals, and 30 were individuals who had visited KSA for Hajj or Umrah. Of the participants, 34 (37.8%) were females and 56 (62.2%) were males. The age range was 22–59 years. Additionally, 90 dromedary camels were also included; 50 (55.6%) males and 40 (44.4%) females. The age range was 1–15 years. Serum samples from subjects were collected and tested for the presence of anti-MERS-CoV IgG using the recombivirus human anti-MERS-CoV spike protein S1 domain (MERS-S1) IgG ELISA kit (Alpha Diagnostic International, USA).

Statistical analysis was done using SPSS (IBM-SPSS), Version 23. Data were presented in simple measures of frequency, percentage, mean, median, and range. The significance of difference of different means (quantitative data) was tested using Student’s t-test for difference between two independent means or ANOVA test for difference among more than two independent means. The significance of difference of different percentages (qualitative data) was tested using Pearson’s chi-squared test (χ2-test) with application of Yate’s correction or Fisher’s exact test whenever applicable. Statistical significance was considered whenever the p-value was equal to or less than 0.05.

## 3. Results

The Shapiro–Wilk test was conducted to check if the normal distribution model fits the observations, and since the sample size is greater than 50, the normal approximation was used to calculate the p-value (a significance level (α) of 0.05 was used). For the human subjects, the Shapiro–Wilk test did not show a significant departure from the normality [W(90) = 0.9731, p = 0.05899]. Similarly, the Shapiro–Wilk test did not show a significant departure from the normality for camels [W(90) = 0.9726, p = 0.0539].

Results revealed that 46 (51.1%) of humans were positive for anti-MERS-CoV IgG, with 95% confidence interval for the prevalence rate 40.9–61.3. Additionally, the interquartile range of anti-MERS-CoV IgG titer was 5–19.7 U/mL with a mean of 81.2 U/mL.

Looking at Table 1, it is clear that there was insignificantly higher MERS-CoV IgG positivity rate and anti-MERS-CoV IgG Ab titer among humans 40–49 years old compared to other age groups (p = 0.08 and p = 0.11, respectively).

The anti-MERS-CoV IgG positivity rate was insignificantly higher among female humans compared to males (61.8% vs 44.6%, p = 0.12), However, the mean, median and interquartile range of anti-MERS-CoV IgG titer were significantly higher among females (p < 0.004) as shown in Table 2.

The results in Table 3 showed that the anti-MERS-CoV IgG positivity rate among people who visited KSA for Hajj or Umrah (93.3%) was significantly higher compared to other groups (p < 0.001).

Likewise, the anti-MERS-CoV IgG Ab titer was significantly higher among people who visited KSA for Hajj or Umrah compared to other groups (p < 0.001).

The results also showed that human participants without symptoms had significantly higher anti-MERS-CoV Ab titer compared to those with one or more symptoms (p = 0.003). The flu-like syndrome was found to be the only symptom significantly associated with high anti-MERS-CoV Ab titer (p = 0.001), while other symptoms including, dry cough, productive cough, sore throat were insignificantly associated with anti-MERS-CoV Ab titer, as demonstrated in Table 4.

In the camels, results revealed that 81 (90.0 %) of camels were positive for anti-MERS-CoV IgG, with 95% confidence interval for the prevalence rate 82.5–94.9. Additionally, the interquartile range of anti-MERS-CoV IgG titer was 1.6–94.3 with a mean of 99.8 U/mL.

In Table 5, it is clearly obvious that the anti-MERS-CoV IgG positivity rate and anti-MERS-CoV IgG titer were insignificantly higher among camels 10–15 years old compared to other age groups (p = 0.88 and p = 0.79, respectively).

The anti-MERS-CoV IgG positivity rate was equally distributed in female and male camels (90.0%, p = 1), but the mean, median, and interquartile range of anti-MERS-CoV IgG titer was insignificantly higher among males compared to females (p = 0.57) as shown in Table 6.

## 4. Discussion

To the best of our knowledge, this cross-sectional study is the first one in Iraq on MERS-CoV infection. Probably the only single limitation is the small sample size of both subjects included, merely because this study was personally funded and the laboratory diagnostic kits were costly.

Certainly, the most fascinating result of this study is the high infection rate of MERS-CoV among Iraqi camels, which also harbor high anti-MERS-CoV IgG titer that certainly reflects the presence of high viral load. Similar results had been obtained from camels in the Middle East, particularly Arabian Peninsula, Africa, and Asia [4,6,9,17]. Additionally, 84.5% of serologically tested camels from Egypt and 91.4% camels from Sudan had MERS-CoV neutralizing antibodies [8]. The importance of these results is streaming from the regional and global scientific consent that dromedary camels are the only animal species for which there were convincing evidences that it is a host species for MERS-CoV and hence a potential source of human infections [24,25].

Geographically, it is well known that Iraq has a long common land borders with KSA, the country where the MERS-CoV was first recognized in 2012, and the single country which reported more than 80% of the MERS-CoV cases worldwide [26]. These borders are mostly deserts in which the camels form the main vehicle across the borders for trading. Earlier epidemiological surveys had affirmed that the MERS-CoV was endemic in Saudi’s camels and that Saudi Arabian dromedary camels show significantly higher MERS-CoV carrier rates than dromedary camels imported from Africa [18]. This high prevalence of infection with MERS-CoV actually was responsible for large human MERS-CoV outbreaks in KSA [1,18,27]. The presence of high anti-MERS-CoV Ab titer among Iraqi camels probably reflects the presence of high viral load and consequently high shedding of the virus with respiratory secretions and thus high infectivity of these camels. Several studies had documented that camels with high viral load were able to shed the MERS-CoV particularly with respiratory secretions for long duration [7,11,24]. Therefore, it is crucial to conduct further studies on Iraqi camels for molecular detection of MERS-coronaviral RNA, and to investigate the infectiousness of these camels.

The results also revealed that there is insignificantly higher positivity rate among camels 10–15 years old plus insignificantly higher anti-MERS-CoV IgG Ab titer among the same age group. This finding was controversial among previous studies [7,16]. However, generally it has been found that MERS-CoV seroprevalence increased with age of camels [12]. Accordingly, the results of this study seem reasonable and acceptable since older camels are usually used for trading across the Iraqi-KSA borders, and hence have more chance to catch the MERS-CoV infection from carrier camels [3], or through importation of camels from endemic areas, and in this regard, it was found that Saudi Arabian dromedary camels had significantly higher MERS-CoV carrier rates than dromedary camels elsewhere [2]. Additionally, younger camels may still have protective levels of maternal anti-MERS-CoV Ab. Therefore, further studies are needed on Iraqi dromedary camels to address these queries.

The present results found that 46 (51.1%) of Iraqi population were seropositive for anti-MERS-CoV IgG, with a mean of 81.2 U/mL of anti-MERS-CoV IgG titer. That is a really high infection rate. Unfortunately, similar studies were scarce in the literature. However, in KSA it has been found that the anti-MERS-CoV antibodies were confirmed in 0.15% of 10,009 people tested in six of the 13 provinces [1,28]. Actually, that necessitates urgent local or country-wide surveillance studies to affirm these data and for setting up effective national control precautions.

Moreover, a significantly higher infection rate was recorded among those people who had previous travel to KSA for Hajj or Umrah (93.3%), plus a significantly higher anti-MERS-CoV IgG Ab titer (mean serum IgG titer was 72 U/mL). Upon reviewing the literature, several countries had denied any infection by MERS-CoV in their citizens after their return form KSA, while other countries affirmed these infections [16,20–22]. More comprehensively, it was found that severe acute respiratory syndrome (SARS) coronavirus and MERS coronavirus were never isolated in Hajj pilgrims, and that the most commonly isolated viruses from symptomatic patients during the Hajj by PCR were rhinovirus (5.9%–48.8%), followed by influenza virus (4.5–13.9%) and non-MERS coronaviruses (2.7%–13.2%) [14]. Probably, this is the most acceptable explanation for the 35% MERS-CoV infection rate among normal healthy population in our study. It is important to know that person-to-person transmission of MERS-CoV is still mostly limited to health care settings [16,25]. Therefore, further studies are needed to verify the present results.

The results also revealed that 27.5% of camel’s contacts (camel’s owners and slaughter house workers) were positive for anti-MERS-CoV IgG with a mean IgG titer of 30.2 U/mL. These results are not unusual as many previous studies had ascertained the central role of camels for transmission of MERS-CoV to human contacts [3,5,10]. An epidemiological study had reported that MERS-CoV human cases without documented contact with another human MERS-CoV case make up 61% (517/853) of all reported cases, and dromedary camels are the only animals that can transmit infection to human [24]. The long duration of viral shedding from infected camels provided further evidence of the zoonotic potential of MERS-CoV infection and strongly suggested that camels may have a role in the transmission of the virus to humans [6]. The present study also found that the anti-MERS-CoV IgG positivity rate and the anti-MERS-CoV IgG Ab titer were insignificantly higher among those who are 4–49 years old. These results are in agreement with those reported by other epidemiological studies in KSA which affirm that the majority of the affected patients were aged ≥40 years [2,15,27].

Regarding the sex, the anti-MERS-CoV IgG positivity rate and the anti-MERS-CoV IgG titer was insignificantly higher among females. Although the results in this respect were controversial, males were predominant [2,15,26]. Clinically, among all symptoms investigated including, dry cough, productive cough, sore throat, and shortness of breath, only flu-like syndrome was significantly associated with high anti-MERS-CoV Ab titer. Additionally, the anti-MERS-CoV IgG titers were significantly higher among asymptomatic participants compared to those with one or more symptoms, since the current infection in symptomatic participants had yet to yield a high Ab titer, whereas asymptomatic participants, most probably, had contracted MERS-CoV previously and developed a high Ab titer ever since. Studies in this regard had yielded variable results. Among patients with confirmed MERS-CoV infection in KSA, 81% were symptomatic at presentation, with fever being the most common complaint [15]. Similar results had been reported by other studies [2,22,27]. It is important to mention that asymptomatic or mild MERS-CoV infection was recognized in UAE and during the Korean outbreak but transmission from these cases was not reported [5,15,29].

## Figures and Tables

**Table 1:** The anti-MERS-CoV IgG positivity rate and titer by age groups in humans.

Age groups (No.)	IgG-positive humans (%)	95% CI for prevalence rate	Serum anti-MERS-CoV IgG titer (U/mL)			
			Range	Median	Interquartile range	Mean rank
< 40 (30)	12 (40)	24–57.8	1.7–83	2.7	1.8–3.9	38.2
40–49 (26)	18 (69.2)	50.2–84.2	1.6–94.1	4.5	2.2–72.6	52.6
50–59 (34)	16 (47.1)	31.1–63.5	1.7–94.3	2.6	2–75.6	46.5
p-value	0.08		0.11			

**Table 2:** The anti-MERS-CoV IgG positivity rate and titer by sex in humans.

Sex (No.)	IgG-positive humans (%)	95% CI for prevalence rate	Serum anti-MERS-CoV IgG titer (U/mL)			
			Range	Median	Interquartile range	Mean rank
Female (34)	21 (61.8)	45–76.6	1.7–94.1	21.6	2.4–73.8	55.6
Male (56)	25 (44.6)	32.2–57.7	1.6–94.3	2.4	1.8–4.45	39.4
p–value	0.12		< 0.004			

**Table 3:** Anti-MERS-CoV IgG positivity rate according to human participant groups.

Participant groups	Total No.	Serum IgG-positive humans (%)	95% CI for prevalence rate
Camels close contacts	40	11 (27.5)	15.6–42.5
Normal healthy individuals	20	7 (35)	17.2–56.8
Hajj or Umrah pilgrims	30	28 (93.3)	80.3–98.6
p-value	< 0.001		

**Table 4:** Anti-MERS-CoV IgG titer (U/mL) by clinical symptoms in humans.

Any symptoms	Total no.	Serum IgG titer (U/mL)				
		Range	Median	Interquartile range	Mean rank	p-value
Negative	37	1.6–94.1	5.0	2.5–75.6	55.2	p = 0.003
Positive	53	1.7–94.3	2.3	1.8–3.9	38.8	
Flu-like symptoms	Total No.	Serum IgG titer (U/mL)				
		Range	Median	Interquartile range	Mean rank	p-value
Negative	68	1.6–94.3	2.3	2.3	37.9	p = 0.001
Positive	22	2–94.1	69.2	69.2	69	

**Table 5:** The anti-MERS-CoV IgG positivity rate and titer by age groups in camels.

Age groups (no.)	IgG positive camels (%)	95% CI for prevalence rate	Serum anti-MERS-CoV IgG titer (U/mL)			
			Range	Median	Interquartile range	Mean rank
< 5 (18)	16 (88.9)	68.9–97.6	0.7–49.3	11.2	4.5–17.6	41.9
5–9 (35)	31 (88.6)	75.1–96	0.8–37.5	12.2	5–20.7	45.8
10–15 (37)	34 (91.9)	79.9–97.7	0.6–55.1	13.1	5.6–20.2	47
p-value	0.88		0.79			

**Table 6:** The anti-MERS-CoV IgG positivity rate and titer by sex in camels.

Sex (No.)	IgG-positive camels (%)	95% CI for prevalence rate	Serum anti-MERS-CoV IgG titer (U/mL)			
			Range	Median	Interquartile range	Mean rank
Female (40)	36 (90%)	78–96.5	0.8–55.1	11.7	4.5–19.2	43.8
Male (50)	45 (90%)	79.5–96.1	0.6–49.3	13.6	5.1–20.7	46.9
p-value	1		0.57			
